# Multiridge Method for Studying Ground-Deformation Sources: Application to Volcanic Environments

**DOI:** 10.1038/s41598-018-31841-4

**Published:** 2018-09-07

**Authors:** R. Castaldo, A. Barone, M. Fedi, P. Tizzani

**Affiliations:** 1 National Research Council (CNR) – Institute for the Electromagnetic Sensing of the Environment (IREA), Via Diocleziano, 328 – 80124 Napoli, Italy; 2 Department of Earth, Environmental and Resources Science, University of Naples “Federico II”. Monte Sant’Angelo (L building), Via Cinthia 21, 80126 Napoli, Italy

## Abstract

Volcanic phenomena are currently monitored by the detection of physical and chemical observations. Generally, the ground deformation field is the most relevant shallow expression of the geometric and physical parameters variations in the magmatic reservoir. In this study, we propose a novel method for the direct estimation of the geometric parameters of sources responsible for volcanic ground deformation detected via the DInSAR technique. Starting with the biharmonic properties of the deformation field, we define an approach based on the Multiridge and ScalFun methods to achieve relevant information about both the positions and shapes of active sources, such as the Mogi source. Our methodology is definitely different from the methods currently used for modeling ground-deformation sources, mainly based on forward or inverse techniques. In fact, (i) it does not require any assumptions about the source type, and (ii) it is not influenced by the distribution of medium elastic parameters or (iii) the presence of high-frequency noise in the dataset. For synthetic cases, we accurately estimate the depth to the source within a 3% error. Finally, we study the real case of the Okmok volcano ground-deformation field and achieve results compatible with those in previous works.

## Introduction

In recent decades, the development of remote sensing technologies has provided relevant efforts in the context of earth science. Specifically, the Differential SAR Interferometry (DInSAR) technique has significantly contributed to the characterization and imaging of the deformation of Earth’s surface, allowing for the analysis of a large variety of geological phenomena, such as landslides^1,2^, earthquakes^3,4^ and volcanic events^5,6^.

In a volcanic environment, the accumulation processes of shallow magma in the crust often cause slight movements at the surface, which can be measured using standard land-surveying techniques (leveling) or satellite geodesy methodologies (e.g., GPS and DInSAR). In this context, these aforementioned processes often result from variations in several geometric and physical parameters of magmatic sources (e.g., shape, size, depth, dip, and magnitude of pressure change Δ*P*)^7^. The estimation of these parameters is performed using two different approaches: (i) forward modeling^8,9^, which is based on a trial-error procedure and strongly depends on the experience of the scientist, and (ii) inverse modeling^10–12^, which is faster than the previous approach. Current methods of inversion are essentially based on optimization techniques, where the final solution is that obtaining the best-fit between the measured and modeled data; this optimization/inversion procedure is performed by using algorithms such as Monte-Carlo, Gibbs, Simulated Annealing, and Levenberg-Marquardt^10,13,14^. In the case of the optimization/inversion of geodetic data, several authors have evaluated model parameters via posterior probability density distributions^15,16^. However, we remark that these methods are often unable to provide unique solution^17^.

Starting with these considerations, we propose a novel method based on the theory of Laplace’s equation:

1 $${\nabla }^{2}V=0,$$

where ${\nabla }^{2}$ represents the Laplace operator and $V$ represents the potential (or any of its any-order derivatives). The solutions to Laplace’s equation, which is the simplest example of elliptic partial differential equations, are the so-called harmonic functions, and the theory is often referred to as the Potential Field Theory^18^ (PFT).

We study the conditions under which the theory of the deformation field reduces to the PFT and then show how to obtain the main geometric parameters of the active source.

The PFT can be involved to analyze the deformation fields by considering Love’s argumentation^19^. We further assume that modeling the magmatic reservoir is performed through the approximation of the elasticity theory^7^ and under the assumption that an active volcanic source is not affected by shape changes^19,20^. Among the sources of deformation, we first consider those that also satisfy the homogeneity equation^18^, such as a Mogi source^21^. In this framework, we use methods that have been applied to analyze potential fields^22^, namely, the Multiridge^23^ and ScalFun^24^ methods. By using these, the geometric features of the active source, such as the position and source type, can be easily detected; the main advantages of the proposed methods are that they are determined independently from (i) other physical features of the source (such as the pressure variation; $\Delta P$), (ii) physical-elastic parameters of the medium (such as the shear modulus, $\mu$ and the Poisson ratio $\nu$), and (iii) a low signal-to-noise ratio.

To show the applicability of the proposed methodology, we first carry out a preliminary analysis on the harmonicity and homogeneity properties of the deformation field, based on the simplest volcanic source. Then, we study several test configurations by analyzing the field components and the satellite Line Of Sight (LOS) projection of the deformation field. Finally, we consider the deformation field of the Okmok volcano (Aleutian Islands), which has already been interpreted with standard methods in other works^12,25–28^.

## Methods: Potential Theory for the Deformation Field

The method is applied to interpret the large number of deformation points achieved by the SBAS-DInSAR technique^29^. In particular, we consider the Multiridge^23^ and ScalFun^24^ methods, which are two well-assessed methods, to analyze homogeneous potential fields. For the first approximation, we assume that the ground deformation field results only from volume changes in subvolcanic bodies (i.e., magma chambers) in a linear elastic medium.

### Harmonic functions

A harmonic function ($V$) can be defined as a function that satisfies Laplace’s equation^18^. In particular, a potential field (e.g., ${\bf{F}}(x,y,z)$) is a vector field where the scalar function $V$ (i.e., the potential function) exists^18^:

2 $${\bf{F}}(x,y,z)=(\frac{\partial V}{\partial x},\frac{\partial V}{\partial y},\frac{\partial V}{\partial z})=\nabla V,$$

where the potential function $V$ is a harmonic function that satisfies Laplace’s equation with its partial derivatives of any order at any point not occupied by the sources of field ${\bf{F}}$:

3 $${\nabla }^{2}V=\frac{{\partial }^{2}}{\partial {x}^{2}}V+\frac{{\partial }^{2}}{\partial {y}^{2}}V+\frac{{\partial }^{2}}{\partial {z}^{2}}V=\nabla \cdot \nabla V=0.$$

The interpretation of the ground deformation field via the PFT-based methods derives from Love’s work on the elasticity theory^19^. According to his arguments, when the modeled deformation field is not caused by a source shape-change, the deformation field ${\bf{u}}$ can be represented by the coefficients of the partial differential equation (PDE), with respect to the coordinates of a single scalar function ($\varphi$)^19^:

4 $${\bf{u}}(x,y,z)=[\begin{array}{c}u\\ v\\ w\end{array}]=(\frac{\partial \varphi }{\partial x},\frac{\partial \varphi }{\partial y},\frac{\partial \varphi }{\partial z})=\nabla \varphi .$$

By combining equation (4) with Navier’s equation, to describe the features of **u** in a homogeneous and isotropic medium under equilibrium conditions:

5 $$(\lambda +\mu )\nabla \nabla \cdot {\bf{u}}+\mu {\nabla }^{2}{\bf{u}}=0,$$

we find that the displacement potential $\varphi$ is a biharmonic function since $\varphi \,\,$satisfies^20^:

6 $${\nabla }^{2}{\nabla }^{2}\varphi ={\nabla }^{4}\varphi =0.$$

This result may be also obtained under the Helmholtz formulation^20^.

We show that the ground deformation field modeled with the Mogi source also encompasses harmonic properties. The deformation field modeled by a hydrostatic pressure change within a spherical cavity embedded in an elastic half-space, with a radius much smaller than its depth^21^, is given by:

7 $${\bf{u}}=(\begin{array}{c}{a}^{3}\Delta P\frac{1-\nu }{\mu }\frac{x-{x}_{0}}{{|{\bf{r}}|}^{3}}\\ {a}^{3}\Delta P\frac{1-\nu }{\mu }\frac{y-{y}_{0}}{{|{\bf{r}}|}^{3}}\\ {a}^{3}\Delta P\frac{1-\nu }{\mu }\frac{z-{z}_{0}}{{|{\bf{r}}|}^{3}}\end{array}),$$

where $({x}_{0},{y}_{0},{z}_{0})$ represents the coordinates for the position of the source center, $a$ the radius of the sphere, $\Delta P$ the variation in pressure, ${z}_{0}$ the depth of the source from the center of the sphere, $\mu$ the shear modulus, $\nu$ the Poisson ratio and $|{\bf{r}}|=\sqrt{{(x-{x}_{0})}^{2}+{(y-{y}_{0})}^{2}+{(z-{z}_{0})}^{2}}$. It is simple to argue that **u** is the gradient of the Newtonian potential in the form 1/*r*, which is a harmonic function^18^. Therefore, all of the components of the deformation field **u** are also harmonic, and we find that:

8 $$\nabla \cdot \nabla {\bf{u}}={\nabla }^{2}{\bf{u}}=0.$$

In the case of the LOS satellite data analysis, the projection of the modeled deformation (${u}_{LOS}$) can be simply calculated by combining the ground deformation field components ($u,v,w$) with the LOS unit vector as follows:

9 $${u}_{los}=u\,{c}_{x}+v\,{c}_{y}+w\,{c}_{z},$$

where ${c}_{x}$, ${c}_{y}$, and ${c}_{z}$ are the components of the LOS vector ${\bf{c}}$. The operation of equation (9) also returns a harmonic function^18^, since we consider the mean values of LOS vector components.

### Multiridge method

The Multiridge^23^ method is a multiscale method based on the analysis of so-called ridges, which are defined as lines passing through the maxima of a field and its derivatives at different scales. We emphasize that this method can only be applied in cases when the field can be expressed by harmonic functions.

We consider a coordinate system where the $x$- and $y$-directions are represented by the North-South and East-West directions, respectively, and the $z$-axis is definite and positive downward. If $P(x,y,z)$ is a generic field generated by a simple source $Q({x}_{0},{y}_{0},{z}_{0})$ and by taking into account the cross section $y={y}_{0}$, it is possible to obtain three equations for the ridges relative to the minima of the horizontal derivative of the field^23^:

10 $$\begin{array}{c}x={x}_{0}\\ x-{x}_{0}=\gamma (z-{z}_{0})\\ x-{x}_{0}=-\,\gamma (z-{z}_{0})\end{array},$$

where $\gamma$ is the angular coefficient of the straight line $\gamma =\,\tan \,\beta$, where $\beta$ represents the angle of the ridge line from the vertical $z$-axis. Since the three ridges intersect at the source center $({x}_{0},{z}_{0})$, its position can be simply individuated with this geometric method.

Specifically, the Multiridge method mainly consists of three phases:

1. The creation of a multiscale dataset by performing upward continuation^18,30^ from the original measurement level to different levels,
2. Individuation and representation of the edges, and
3. Representation and continuation of the ridges down to the source-region, to individuate the correct depth of the source at the intersection of more ridges.

Regarding the application of the upward continuation operator to the deformation field (1), we specify that we are clearly not considering that the deformation field could propagate into the air. The upward-continued deformation field instead corresponds to that which would have been produced by the same source in a region that was upper extended by the same amount of upward continuation. For example, if the analyzed field is observed by a source with a depth of ${z}_{0}=2\,\,$km, the upward-continued field towards a 1-km altitude is generated by the same source but with a center depth at ${z}_{0}=3$ km. The scientific soundness of this procedure is shown in the Supplementary Information (Figure S1).

For the concerns of this study (2), we clarify that more than one subset can be defined: a multiridge subset, ${R}_{1}$, that is individuated by the zeros of the horizontal derivatives; a multiridge subset, ${R}_{2}$, that is individuated by the zeros of the first-order vertical derivative; and a multiridge subset, ${R}_{3}$, that is individuated by the maxima of the field^22^. In our analysis, we have selected the ridges of subsets ${R}_{1}$ and ${R}_{2}$. Moreover, since the method involves the field at different levels (i.e., multiscale), high-frequency noise can be easily recognized in the multiridge subsets. For this reason, in the real-case data analysis, we have to exclude the edges related to noise.

For the representation and continuation of the ridges (3), we specify that each ridge is determined by a best-fit linear regression within a 95% confidence interval; in particular, we calculate the determination coefficient R^2^, where R is the correlation coefficient, which represents a statistical measure of how the data (edges) are close to the fitted regression line (ridges). Moreover, we evaluate the solution uncertainties (intersection at the ridges) by considering the error on the best-fit linear regression coefficients (intercept and slope constants).

We remark that in the case of irregular (not flat) data measurement levels, we have to perform another procedure before applying the Multiridge method. Since the method is based on a level-to-level algorithm, and the ground deformation field is measured at the topographic surface (which is generally detailed), the dataset must be processed to numerically generate a ground deformation field that could have been measured in the case of constant distance between the source and measurement surface. This transformation relocates the analyzed field onto the constant reduction level, which is performed in this study by using the CWT-domain algorithm^31^. The accuracies and details of this procedure are shown in the Supplementary Information (Figures S2 and S3).

### Homogeneous functions

Consider again the ground deformation field modeled through the Mogi source, and let us evaluate its homogeneity properties.

The function $U$ is said to be homogeneous, with degree $n$, if satisfies the Euler’s equation^18^:

11 $$x\frac{\partial U}{\partial x}+y\frac{\partial U}{\partial y}+z\frac{\partial U}{\partial z}=nU.$$

The importance of the homogeneous functions derives from the fact that simple source fields are homogeneous functions of degree $n$ which, for many sources, reflects the falloff rate of the potential field anomaly with distance^24^. By inserting equation (11) into equation (7), it is obvious that each component of ${\bf{u}}$ for Mogi’s source model is homogeneous of degree $n=-\,2$. The homogeneity degree of the field ($n$) may be used to estimate the homogeneity degree of the source (${n}_{s}$)^32^ which, for a Mogi model, is given by:

12 $${n}_{s}=n-1.$$

Since ${n}_{s}\,\,$is a source parameter determined by a field parameter ($n$), it may be convenient to refer to its opposite, the so-called Structural Index ($N$):

13 $$N=-\,{n}_{s},$$

which is a parameter utilized within the framework of homogeneous functions to study gravity and magnetic field anomalies^33,34^. We conclude that the Mogi model source is characterized by $\,{n}_{s}=\,-3$ and $N=3.$

### ScalFun method

The ScalFun method is based on the properties of the scaling function, which was introduced into the framework of the DEXP theory^24^ to estimate the homogeneity degree of the observed field ($n$); this is, in turn, related to the homogeneous property of the source (${n}_{s}$). For any ${p}^{th}$ vertical derivative for the Newtonian potential of a pole source ${f}_{p}(z)\,\,$at $x={x}_{0}$ and $y={y}_{0}$, we define the scaling function ${\tau }_{p}$ of order $p$ as:

14 $${\tau }_{p}=\frac{\partial \,\mathrm{log}({f}_{p}(z))}{\partial \,\mathrm{log}(z)}=-\frac{(p+1)z}{z-{z}_{0}},$$

where $n=-\,(p+1)$ represents the degree of homogeneity of ${f}_{p}$.

Equation (14) can be rewritten by $\,z=\frac{1}{q}$; therefore, ${\tau }_{p}$ becomes a function of $q$:

15 $${\tau }_{p}(q)=\frac{n}{1-{z}_{0}q},$$

which means that ${\tau }_{p}(q\to 0)=n.$

Hence, in a plot diagram of ${\tau }_{p}$ as function of $q$, the intercept gives an estimate of the homogeneity degree $n$. Starting from the ${z}_{0}\,\,$source depth retrieved by using the Multiridge method, we can use equation (15) to estimate $n$, ${n}_{s}\,\,$and *N*; these values give us information about the geometry of the source.

## Result I: Application of the Potential Theory to the Synthetic Deformation Field

To show the validity of the proposed method, we perform several synthetic tests based on the analysis of the deformation field generated by a spherical source. We chose Cartesian reference system, with the origin of the system located at the point ${\bf{O}}(0,0)$; the $x$- and $y$-axes are oriented in the E-W and N-S directions, respectively, and the $z$-axis is negative downward. For all of the following tests, the deformation field is simulated with a grid of 120 km x 120 km sampled at 0.1 km intervals.

### Analysis of the vertical component of the deformation field

**Figure 1:**
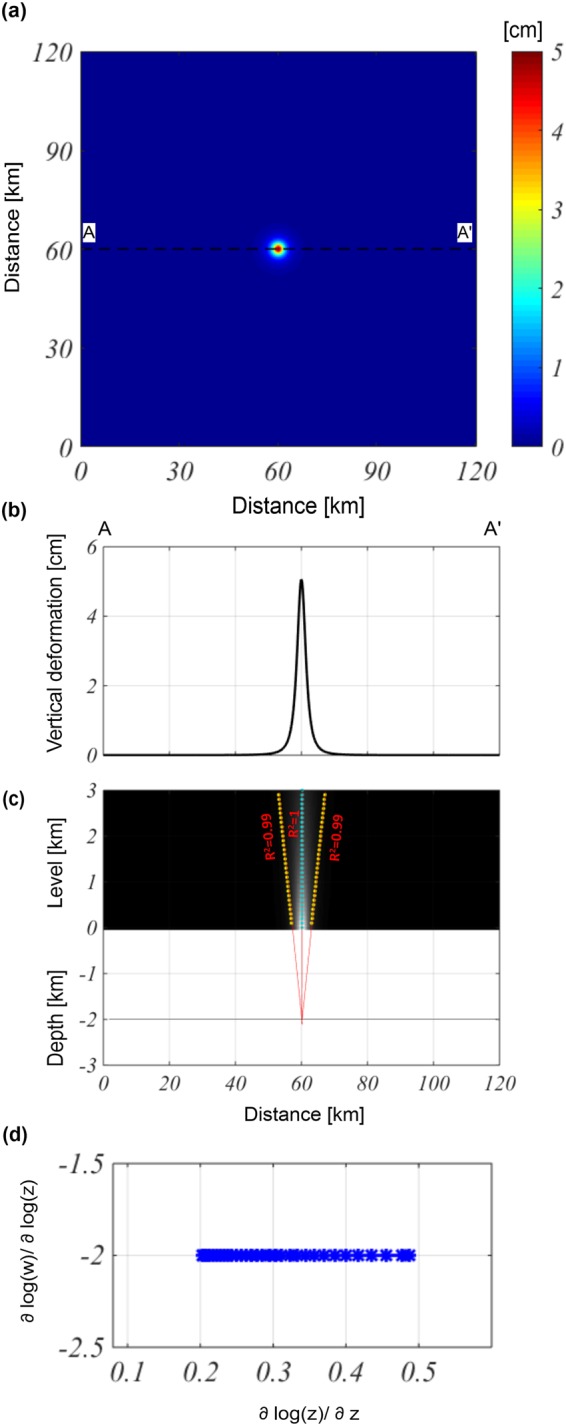
Multiridge and ScalFun methods: the Mogi model vertical component. (**a**) Map of the modeled vertical component of the deformation field calculated on a flat surface level of $z=0$. The black dashed line indicates the AA’ trace. (**b**) AA’ profile of the vertical component of the deformation field; (**c**) The results of the Multiridge method applied to the AA’ noise profile; the red solid lines, which represent the regression lines, estimate the source position at their intersection, and the black solid line indicates the real source depth. For each regression line we indicate the coefficient of determination (R^2^); (**d**) the results of the ScalFun method applied to the central cyan multiridge subset reported in (c), where $w$ represents the values of the field corresponding to the selected ridge.

We generate the vertical component of the ground deformation field on a flat surface ($$z=0)$$ by using the Mogi model, as produced by the overpressured ($${\rm{\Delta }}P=10$$ MPa) spherical magma chamber (Fig. 1a). The active source depth is −2 km and is located 60 km along both the $$x$$- and $$y$$-directions; its radius is 0.3 km, while the medium shear modulus and Poisson’s coefficient are 1 GPa and 0.25 [-], respectively. We apply the Multiridge method to the vertical component, analyze the AA’ E-W profile passing for the maximum value of the field (Fig. 1b). The results allow us to easily identify a source at an approximate −2.05 ± 0.01 km depth and located at 60 ± 0.02 km along the $$x$$-direction (Fig. 1c). The estimation reliability is supported by the high values of R^2^. Then, we apply the ScalFun method to the central ridge (cyan) and achieve a homogeneity degree of $$n\approx -2$$ (Fig. 1d). This value corresponds to the Structural Index value of $$N=3$$, suggesting that the representative geometry of the source is spherical^32^, which is in agreement with the features of the true source.

**Figure 2:**
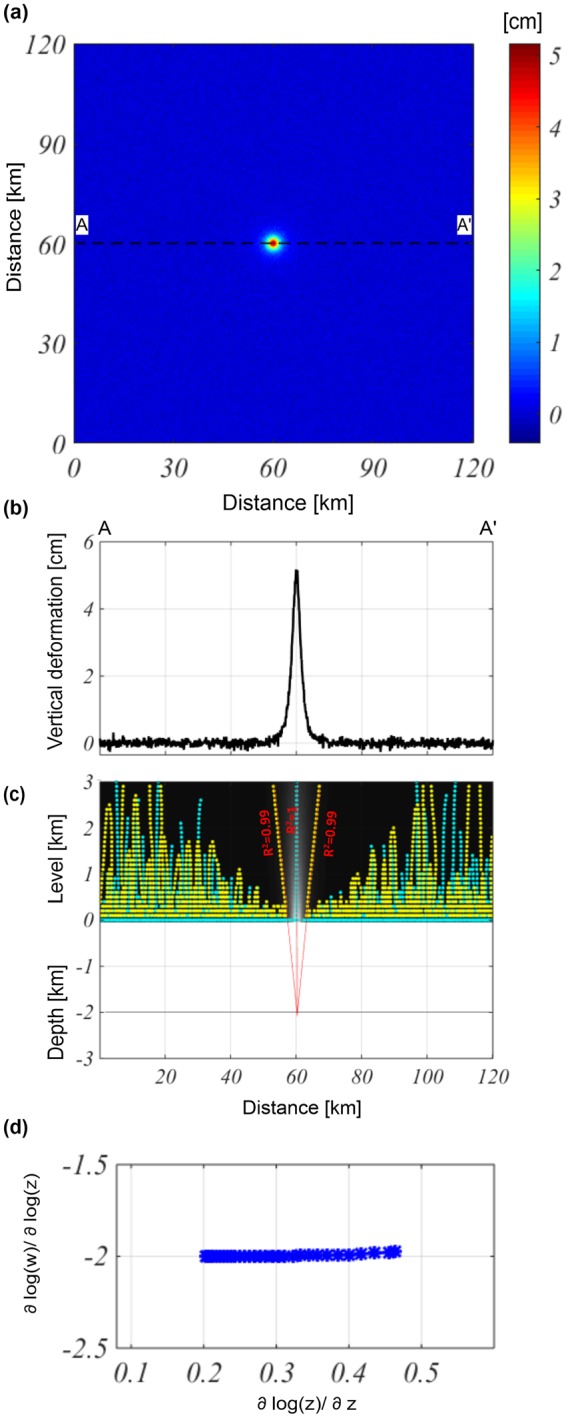
Multiridge and ScalFun methods: the Mogi model vertical component with noise. (**a**) Map of the modeled vertical component of the deformation field calculated on a flat surface level of $z=0$ and perturbed by 10% (with respect to the maximum value) of high-frequency noise. The black dashed line indicates the AA’ trace. (**b**) AA’ profile of the vertical component of the noise deformation field. (**c**) The results of the Multiridge method applied to the AA’ noise profile; the red solid lines, which represent the regression lines, estimate the source position at their intersection, and the black solid line indicates the real source depth. For each regression line we indicate the coefficient of determination (R^2^); (**d**) the results of the ScalFun method applied to the central cyan multiridge subset reported in (c), where $w$ represents the values of the field corresponding to the selected ridge.

Subsequently, we consider the previous test adding to the modeled vertical component a 10% of high-frequency noise (with respect to the maximum value of the anomaly) (Fig. 2a,b). The results show that the estimates are not conditioned by the lower signal-to-noise ratio since (also in this case) we identify a source depth of approximately −2.04 ± 0.02 km, and a location of 60 ± 0.03 km along the $x$-direction (Fig. 2c). Then, we apply the ScalFun method to the central ridge (cyan) in order to evaluate the homogeneity degree of the field, $n\approx -\,2$ ($N=3$) which, in turn, gives us an evaluation of the geometric shape of the source (Fig. 2d).

**Figure 3:**
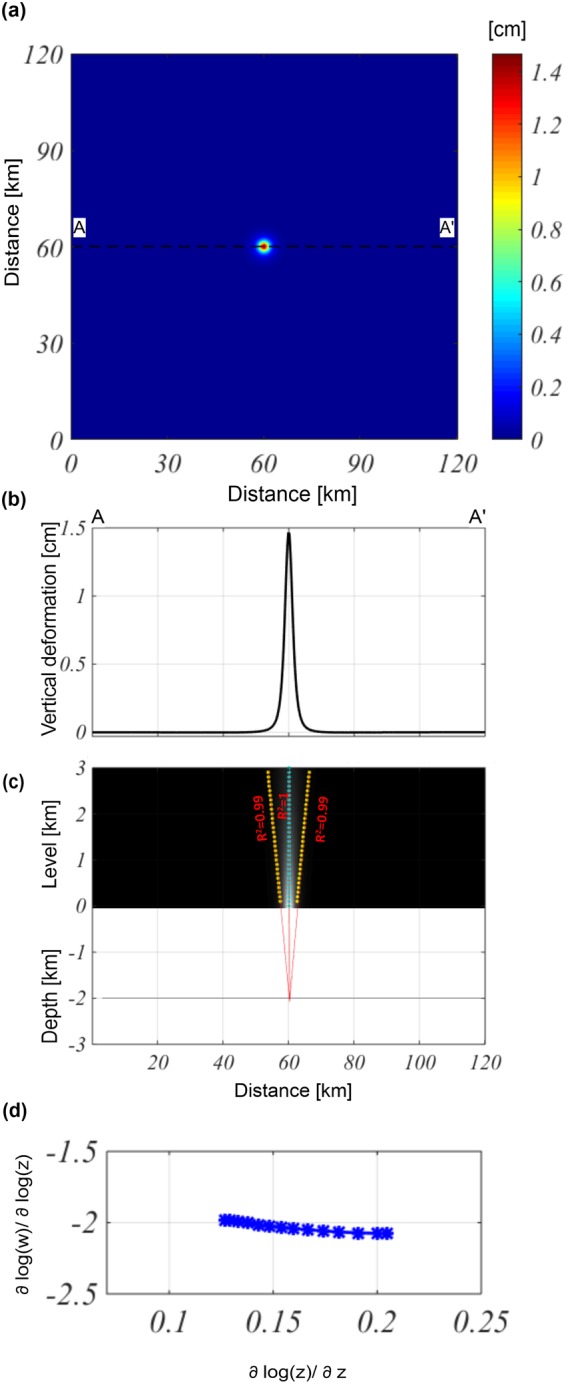
Multiridge and ScalFun methods: spherical source embedded in a heterogeneous medium. (**a**) Map of the modeled vertical component of the deformation field calculated on a flat surface level of $\,z=0$. The black dashed line indicates the AA’ trace. (**b**) AA’ profile of the vertical component of the deformation field. (**c**) The results of the Multiridge method applied to the AA’ noise profile; the red solid lines, which represent the regression lines, estimate the source position at their intersection, and the black solid line indicates the real source depth. For each regression line we indicate the coefficient of determination (R^2^); (**d**) the results of the ScalFun method applied to the central cyan multiridge subset reported in (**c**), where $w$ represents the values of the field corresponding to the selected ridge.

For the third case study, we consider the same source model parameters as those of the previous tests, but it is embedded in a heterogeneous elastic space. In particular, using a 3D finite element approach, we run the model by considering the heterogeneities of the elastic parameters (increasing shear modulus with depth) of the medium (Figure S4). The modeled vertical component is reported in Fig. 3a. By analyzing the AA’ profile (Fig. 3b), we retrieve a source depth of approximately −2.03 ± 0.04 km, and a location of 60 ± 0.05 km along the $x$-direction (Fig. 3c). The application of the ScalFun method confirms the expected result for a Structural Index equal to $\,N\approx 3$ (Fig. 3d).

### Analysis of the LOS deformation field

**Figure 4:**
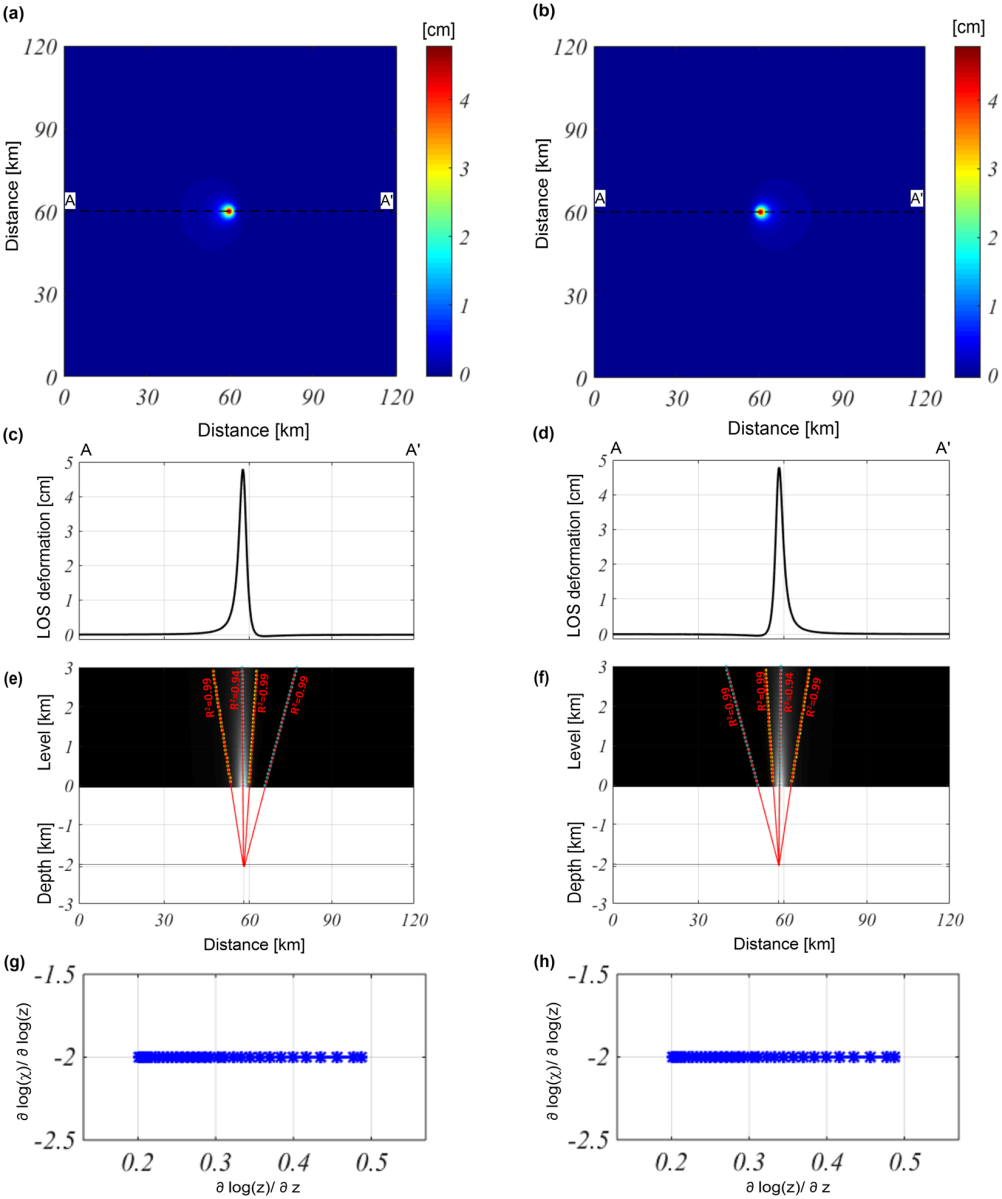
Multiridge and ScalFun methods: LOS-projected Mogi model. (**a**,**b**) Modeled LOS deformation maps projected along ascending and descending orbits, respectively; the fields are calculated on a flat measurement level of $z=0$. The black dashed lines indicate the positions of the AA’ traces. (**c**,**d**) LOS deformation profiles evaluated along the AA’ traces for both the ascending and descending orbits. (**e**,**f**) The results of the Multiridge method applied to the AA’ profiles; (**c**) the red solid lines, which represent the regression lines, estimate the source position at their intersection, and the black solid line indicates the real source depth. For each regression line we indicate the coefficient of determination (R^2^); (**g,h**) the results of the ScalFun method applied to the central cyan multiridge subsets reported in (**e**,**f**) for ascending and descending orbits, respectively. The variable $\chi$ represents the value of the field corresponding to the selected ridges.

**Figure 5:**
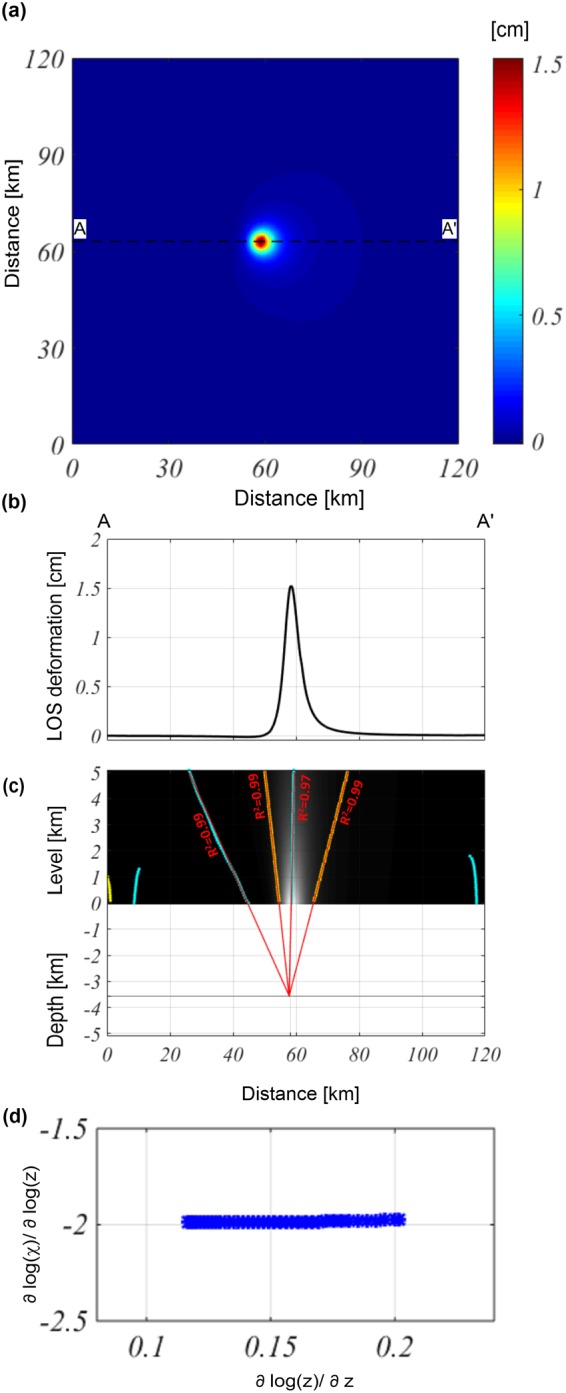
Multiridge and ScalFun methods: LOS-projected Mogi model evaluated at a constant reduction level. (**a**) Descending LOS modeled deformation map retrieved on a 1.5-km flat surface a.s.l.; the original data level is represented by the Okmok volcano topography. The black dashed line indicates the position of the AA’ trace. (**b**) The LOS deformation profile evaluated along the AA’ trace. (**c**) The results of the Multiridge method applied to the AA’ noise profile; the red solid lines, which represent the regression lines, estimate the source position at their intersection, and the black solid line indicates the real source depth. For each regression line we indicate the coefficient of determination (R^2^); (**d**) the results of the ScalFun method applied to the central cyan multiridge subset reported in (**c**), where $\chi$ represents the value of the field corresponding to the selected ridge.

**Figure 6:**
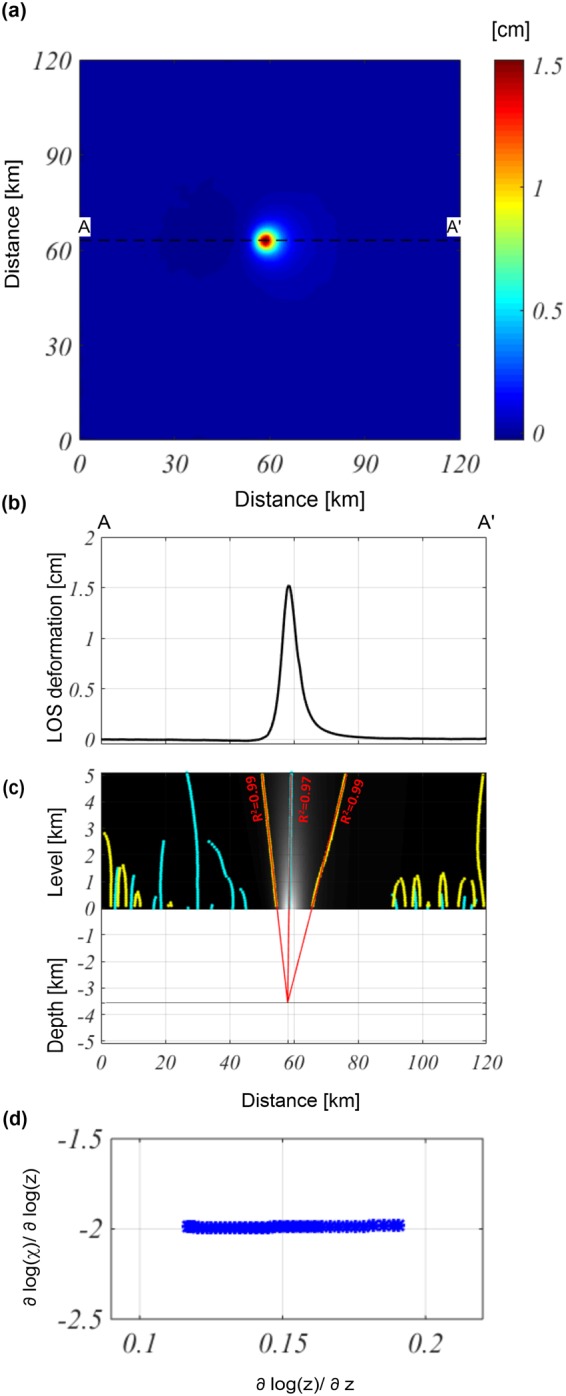
Multiridge and ScalFun methods: the LOS-projected Mogi model evaluated at a constant reduction level with noise. (**a**) Descending LOS modeled deformation map perturbed by 10% (with respect to the maximum value) of the high-frequency noise and retrieved on a 1.5-km flat surface a.s.l.; the original data level is represented by the Okmok volcano topography. The black dashed line indicates the position of the AA’ trace. (**b**) LOS deformation profile evaluated along the AA’ trace. (**c**) The results of the Multiridge method applied to the AA’ noise profile; the red solid lines, which represent the regression lines, estimate the source position at their intersection, and the black solid line indicates the real source depth. For each regression line we indicate the coefficient of determination (R^2^); (**d**) the results of the ScalFun method applied to the central cyan multiridge subset reported in (**c**), where $\chi$ represents the value of the field corresponding to the selected ridge.

We further apply our methodology to the modeled LOS deformation field, which is calculated first on a flat surface at $z=0$ (Fig. 4); then, the Okmok topographic surface is considered (Fig. 5**)** and, finally, the high-frequency noise is added (Fig. 6). All of the synthetic fields are projected along the ascending and descending orbits of the LOS satellite sensor, and we consider a look-angle of 23°.

We consider the same source model parameters as those of the first test, changing only the horizontal position beneath the caldera floor: 58 km in the $x$-direction and 53 km in the $y$-direction. By combining the simulated components of the ground deformation field with the LOS unit vectors ([−0.346, −0.081, 0.935]: mean values for ascending orbit; [0.346, −0.081, 0.935]: mean values for descending orbit), we obtain the projected field along the ascending (Fig. 4a–c) and descending (Fig. 4b–d) orbits. In both cases, the retrieved source depth is approximately −2.05 ± 0.02 km, and the location equal to 58.02 ± 0.02 km along the $x$-direction (Fig. 4e,f). The application of the ScalFun method to both the ascending and descending cases confirms the expected value of the Structural Index ($N=3$) (Fig. 4g,h).

Then, we consider the previous test configuration projected along the descending LOS by changing only the surface measurements from $z=0$ to $z$ equal to the Okmok volcano topography. We process the simulated dataset to relocate the analyzed field at the new constant reduction level, which is chosen to be 1.5-km a.s.l. After this transformation, the expected depth is equal to −3.5 km (i.e., the distance between the constant reduction level and source depth). We apply the Multiridge and ScalFun methods to the processed field shown in Fig. 5a by considering the AA’ profile (Fig. 5b): the first method allows us to identify the source at an approximate −3.53 ± 0.02 km depth (Fig. 5c), and the horizontal position at 58.01 ± 0.02 km along the $x$-direction; the second method reveals the homogeneity degree of the field, $n=-\,2$ (Fig. 5d). This value corresponds to a source with a Structural Index of $N=3$, suggesting that the source geometry is well represented by a spherical geometry.

Finally, we consider the aforementioned test configuration, with 10% high-frequency noise (with respect to the maximum value), on the descending LOS deformation field (Fig. 6a,b). The achieved results confirm that the estimated geometric parameters of the source (−3.55 ± 0.04 km for depth and 58.01 ± 0.02 km for horizontal position) are not influenced by the presence of high-frequency noise in the dataset (Fig. 6c,d).

We point out that for all of the performed tests summarized in Table 1, the same parameter uncertainties have also been achieved by analyzing the N-S profile.

**Table 1:** Synthetic test configuration and estimated parameter uncertainties.

Test	Data	Model Setting	Uncertainties at x_0_, y_0_ [km]	Uncertainties at z_0_ [km]*
1	Vertical component	Hom	60 (± 0.02)	−2.05 ± 0.01
2	Vertical component	Hom + Noise	60 (± 0.03)	−2.04 ± 0.02
3	Vertical component	Het	60 (± 0.05)	−2.03 ± 0.04
4	LOS-ascending; LOS-descending	Hom	58.02 (± 0.02)	−2.05 ± 0.02
5	LOS-descending	Hom + Topo	58.01 (± 0.02)	−2.03 ± 0.02*
6	LOS-descending	Hom + Topo + Noise	58.01 (± 0.02)	−2.05 ± 0.04*

*With respect to the constant reduction level.

## Result II: Application of the Potential Theory to the Real Deformation Field

### Geological setting of the Okmok volcano

The Okmok volcano is an active caldera field located on the oceanic crust of the central Aleutian arc (Alaska – USA)^35^ that represents the surface expression for the subduction of the Pacific Plate as it moves northwards beneath the North American Plate^36^.

In particular, it is a dominantly a basaltic shield volcano covering most of the northeastern end of Umnak Island in Alaska^26^. The Okmok physiography is dominated by a central caldera, with a diameter of 10 km; the rim and caldera floor have elevations of approximately 900 and 400 m a.s.l., respectively^28^. This physiography is the result of two different and large (≈15 km^3^) caldera-forming events^35^ caused by catastrophic pyroclastic eruptions that occurred approximately 12.0 and 2.05 kyr ago, respectively^35,37^. These eruptions began with a small Plinian rhyodacite event, followed by the emplacement of a dominantly andesitic ash-flow unit, whereas effusive inter- and post-caldera lavas have been more basaltic^35^.

Subsequent eruptions produced a field of small cones, which was once entirely filled with water, and lava flows, including several historically active vents. Observed pillow lavas and other textures are consistent with the subaerial eruptions within the caldera^38^.

For the past 200 years, the Okmok volcano has had an effusive and basaltic eruption every 10–20 years, generally from the intracaldera cones^38^. The most recent eruption, in 2008, originated from several new vents and occurred near the eastern rim of the caldera^28^, while the three previous eruptions (1945, 1958 and 1997) originated near the southwest rim of the caldera^39^. In particular, the 1997 eruption began with steam and ash plumes and progressed into moderate Strombolian activity, producing explosive ash plumes and lava that flowed toward the center of the caldera. Geochemical analyses of the erupted products are consistent with the primitive magma from the depth and brief storage of the shallow reservoirs^35^.

### Analysis of the DInSAR measurements

We analyze the Okmok volcano ground deformation pattern retrieved by processing the ENVISAT SAR images. The interferogram is related to the period 15 July 2003–29 June 2004, with images acquired by the ENVISAT satellite (ESA) along the descending orbit (Track 115). The ENVISAT dataset was processed by using the online P-SBAS web tool available within ESA’s Grid Processing On Demand (G-POD) environment, which is within the framework of ESA’s Geohazards Exploitation Platform (GEP)^40,41^. The P-SBAS results from the ENVISAT data were spatially averaged (i.e., multilooked) to obtain a pixel size of approximately 80 m by 80 m on the ground. The ENVISAT satellite look angle is 23°, and the mean values of the LOS unit vector are [0.346, −0.081, 0.935] relative to the satellite descending orbit.

**Figure 7:**
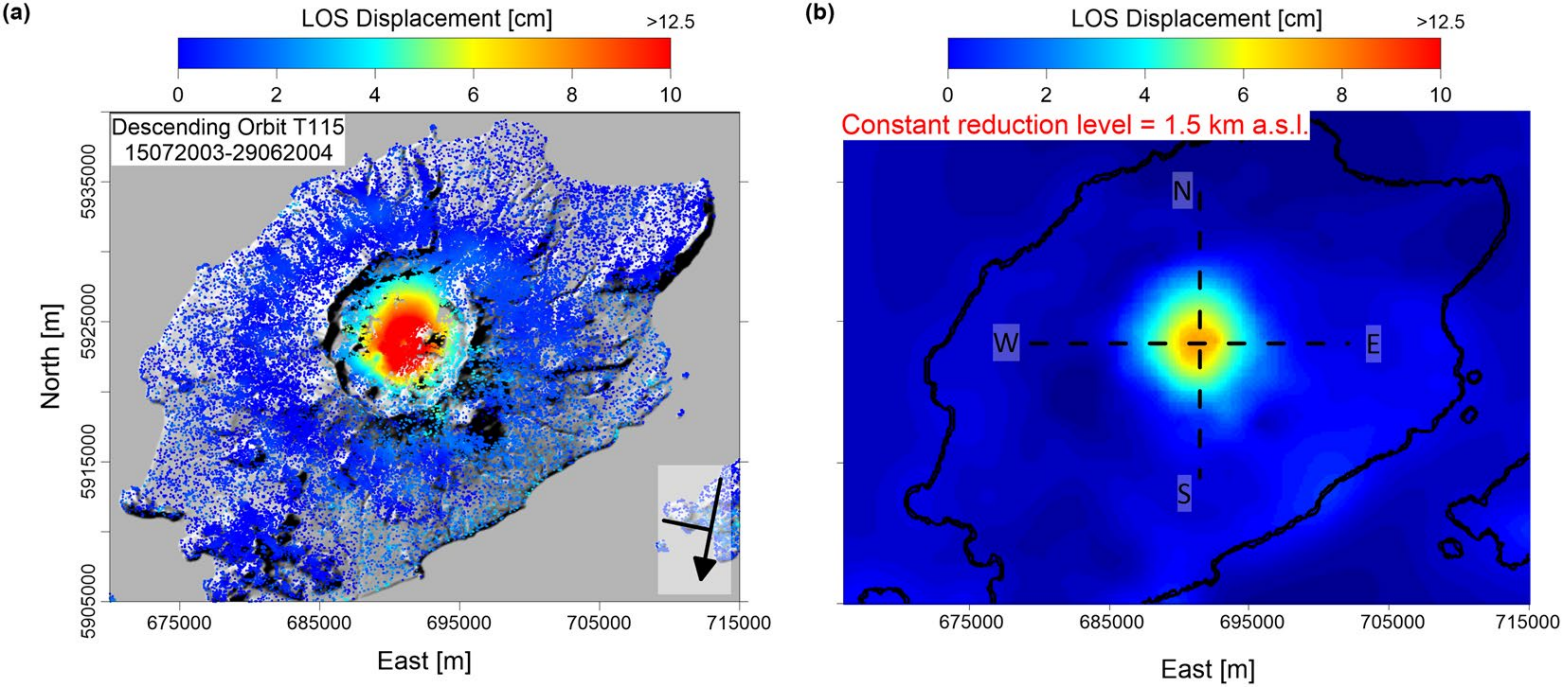
Okmok DInSAR measurements. (**a**) Descending LOS deformation map between 15 July 2003 and 29 June 2004, which is superimposed onto the Okmok volcano topography. The map is generated by using Surfer software (http://www.goldensoftware.com/products/surfer). (**b**) Descending LOS deformation map reduced to a flat surface, which is located 1.5-km a.s.l. The black dashed lines indicate the positions of the E-W and N-S profiles, while the black continuous line represents the island coastline. The map is generated by using Matlab software (https://it.mathworks.com/products/matlab).

After the processing steps, the descending interferogram is unwrapped to retrieve the LOS deformation measurements (Fig. 7a). In the considered period, the DInSAR measurements shown an uplift phenomenon, with a maximum deformation value of approximately 12 cm. Since the measurement surface is detailed (i.e., the Okmok volcano topography), the LOS deformation dataset is processed to obtain the ground deformation field evaluated at the constant reduction level, specifically at 1.5-km a.s.l. (Fig. 7b).

**Figure 8:**
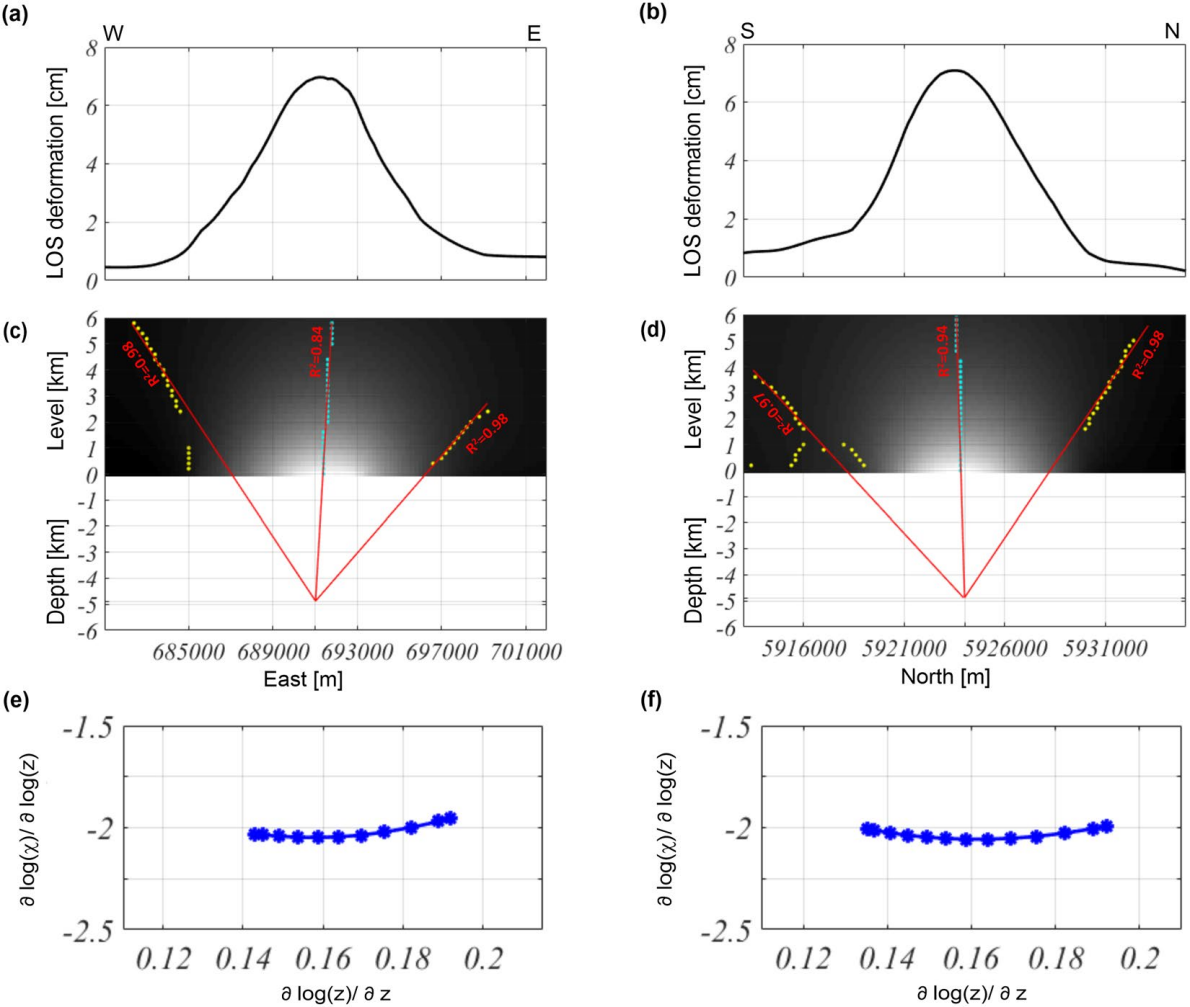
Multiridge and ScalFun methods: the Okmok volcano case. (**a**,**b**) LOS deformation in the E-W and N-S profiles evaluated from Fig. 7(b). (**c**,**d**) The results of the Multiridge method applied to the E-W and N-S profiles; (**c**) the red solid lines, which represent the regression lines, estimate the source position at their intersection, and the black solid line indicates the real source depth. For each regression line we indicate the coefficient of determination (R^2^); (**e**,**f**) the results of the ScalFun method applied to the central cyan multiridge subsets reported in (**c**,**d**), where $\chi$ represents the value of the field corresponding to the selected ridge.

At this stage, we applied our methodology to the DInSAR measurements to evaluate the geometric parameters of the active source; we applied both the Multiridge and ScalFun methods along the E-W (Fig. 8a) and N-S (Fig. 8b) profiles to retrieve the following results:

1. Multiridge method: A source was located at a −4.9 ± 0.06 km depth (Fig. 8c,d) from the constant reduction level (1.5-km a.s.l.) along the *z*-axis, which corresponded to a depth of −3.4 ± 0.06 km b.s.l. with horizontal UTM coordinates equal to 690.9 ± 0.08 km East (Fig. 8c) and 5924 ± 0.07 km North (Fig. 8d);
2. ScalFun: A homogeneity degree of $n\approx -\,2$ (Fig. 8e,f) was computed on the central cyan ridge for both the E-W and N-S profiles; this value corresponds to a Structural Index of $N\approx 3$, suggesting that the source geometry related to the measured ground deformation field can be well-approximated by a spherical reservoir.

## Discussion and Conclusion

In this work, we have proposed and validated a novel method based on the PFT to study the volcanic environment and estimate the geometric parameters of the source model responsible for the ground deformation field.

Multiridge and ScalFun methods provide information on depth, horizontal position and source shape without any a priori information; it is also simple, fast and not computationally expensive. This feature makes it very different from current methods, which are usually based on optimization/inversion procedures and that, most of times, assuming a source-model.

First, we study the harmonicity and homogeneity properties of the theoretical deformation field generated by the Mogi model. We show relevant results since the model equations are simply harmonic and homogeneous functions, with a homogeneous degree of the field of $n=-\,2$, which corresponds to a Structural Index value of $N=3$. These findings are valid for all deformation components (both horizontal and vertical) and LOS projections of the ground deformation field; therefore, this methodology may provide information increasing the reliability of the results. Although this study is limited to the analysis of a spherical source, it shows that when retrieving for $N=3$, the only source responsible for the ground deformation field is a spherical source. In the future, other more complex geometries of deformation sources will be analyzed.

Then, to show the applicability of the Multiridge and ScalFun methods, we have performed several synthetic tests (Table 1) by considering different model configurations. The geometric parameter results show a very good performance of the methodology, with an uncertainty in the estimated source position of approximately 3% respect to the expected value. In all cases, the ScalFun method retrieves the expected source type by estimating a Structural Index of $N=3$. The reliability of the solutions is supported by the high values of R^2^ calculated by the linear regression of each ridge. The achieved uncertainties (Table 1) increase with the complexity of the model setting. In particular, the best solutions are retrieved when the vertical component of the deformation field is simulated on a flat surface, while the worst solution when the deformation field is LOS projected and evaluated on real topography with high-frequency noise.

All of the achieved results emphasize that the proposed methods provide solutions that do not depend on the following: different model pressure changes, ${\rm{\Delta }}P$; the physical-elastic features of the medium in which the source is embedded (e.g., the shear modulus, $\mu$, and Poisson ratio, $\nu$); and the presence of high-frequency noise in the analyzed dataset. On the other hand, the goodness of fit of the results is influenced by the sampling of deformation anomalies (such as the density of the measurement points) and the entirety of their measurements.

Finally, we apply the proposed methodology to the real case of the Okmok volcano ground deformation pattern retrieved by the DInSAR analysis. In particular, we use the interferogram related to the period 15 July 2003 – 29 June 2004, with images acquired by the ENVISAT satellite along the descending orbit. The Okmok volcano deformation field has been studied by many authors^12,26–28^, which all interpreted that deformation was due to the inflation or deflation of a spherical magma chamber. In most of these studies, the source model type (i.e., Mogi model) was a priori assumed, and the elastic parameters (i.e., shear modulus and Poisson ratio) were fixed before inverting the data. Only in one case^28^ did the authors use seismic tomography as a priori information to set the heterogeneous distribution of the elastic parameters. For these authors, the source depth ranged from 3.1 to 3.5 km, while the source position ranged from 690.3 km to 690.72 km for the East UTM coordinate and 5923.6 km to 5923.98 km for the North UTM coordinate (Table 2).

**Table 2:** Source locations for the Okmok magma chamber retrieved from the DInSAR measurements.

Study	Period	East [km]	North [km]	Depth* [km]
*Zhong Lu et al., 2005*	1992–2003	690.55	5923.85	−3.2
*Masterlark et al., 2010*	1995–1997	690.72	5923.98	−3.11
*Biggs et al., 2010*	1992–2008	690.30	5923.60	−3.4
*Masterlark et al., 2012*	1995–1997	690.70	5923.91	−3.52
*This study*	2003–2004	690.90	5924.00	−3.4

*Depths are below sea level.

Our results are in good agreement with those of the aforementioned works, indicating a depth source equal to 3.4 ± 0.06 km and a horizontal position at 690.9 ± 0.08 km E and 5924 ± 0.07 km N (Table 2). Moreover, we estimated a Structural Index of $N=3$ and, therefore, we can state that the geodetic source geometry is well represented by the spherical model. Additionally, for the Okmok real case, we calculated the R^2^ of the best-fit linear regression for each ridge to support the robustness of the proposed solutions. These values are not as high as those retrieved for the synthetic tests, which is probably due to the resolution and sampling step of the measurements. This limitation may be overcome with the measurements retrieved by the new generation of satellite images (e.g., Sentinel-1A and -1B).

In conclusion, since our method does not depend on physical model parameters, we need to apply a subsequent optimization/inversion procedure to fully interpret the ground deformation measurements and determine the source pressure variation, $\Delta P$, and potentially the elastic parameters (such as the shear modulus, $\mu$, and Poisson ratio, $\nu$) of the medium. In the meantime, we stress that the source parameters estimated with the PFT procedure, depth to the center, horizontal position and source-type are decisive to constrain the entire interpretation procedure.

## Electronic supplementary material

Supplementary information

## References

[1] Refice, A., Bovenga, F., Wasowski, J. &Guerriero, L. Use of InSAR Data for Landslide Monitoring: a Case Study from Southern Italy. Geos. and Rem. Sen. Symp. Proceedings. IGARS 2000. IEEE 2000 International (2000).

[2] De Novellis V, Castaldo R, Lollino P, Manunta M, Tizzani P (2016). Advanced three-dimensional finite element modeling of a slow landslide through the exploitation of DInSAR measurements and *in situ* surveys. Remote Sens..

[3] Meyer B (1998). Results from combining tectonic observations and SAR interferometry for the 1995 Grevena earthquake: a summary. J. Geodynamics.

[4] Lavecchia G (2016). Ground deformation and source geometry of the 24 August 2016 Amatrice earthquake (Central Italy) investigated through analytical and numerical modeling of DInSAR measurements and structural-geological data. Geophys. Res. Lett..

[5] Avallone A, Zollo A, Briole P, Delacourt C, Beauducel F (1999). Subsidence of Campi Flegrei (Italy) detected by SAR interferometry. Geoph. Res. Let..

[6] D’Auria L (2015). Magma injection beneath the urban area of Naples: a new mechanism for the 2012-2013 volcanic unrest at Campi Flegrei caldera. Sci. Rep..

[7] Lisowski, M. Anaytical volcano deformation source models. Chapter of Volcano deformation (D. Dzurisin), Praxis Publishing, Chichester, UK (2007).

[8] Lu Z, Mann D, Freymueller J (1998). Satellite radar interferometry measures deformation at Okmok Volcano. Eos.

[9] Lu Z, Mann D, Freymueller J, Meyer DJ (2000). Synthetic aperture radar interferometry at Okmok volcano, Alaska: Radar observations. J. Geophys. Res..

[10] Cervelli P, Murray MH, Segall P, Aoki Y, Kato T (2001). Estimating source parameters from deformation data, with an application to the March 1997 earthquake swarm off the Izu Peninsula, Japan. J. Geophys. Res..

[11] Battaglia M, Troise C, Obrizzo F, Pingue F, De Natale G (2006). Evidence for fluid migration as the source of deformation at Campi Flegrei caldera (Italy). Geophys. Res. Lett..

[12] Biggs J, Lu Z, Fournier T, Freymueller JT (2010). Magma flux at Okmok Volcano, Alaska, from a joint inversion of continuous GPS, campaign GPS, and interferometric synthetic aperture radar. J. Geophys. Res..

[13] Jonsson S (1999). A shallow-dipping dike fed the 1995 flank eruption at Fernandina volcano, Galapagos, observed by satellite radar interferometry. Geophys. Res. Lett..

[14] Tizzani P (2009). Uplift and magma intrusion at Long Valley caldera from InSAR and gravity measurements. Geology.

[15] Brooks AB, Frazer N (2005). Importance reweighting reduces dependence on temperature in Gibbs samplers: an application to the coseismic geodetic inverse problem. Geophys. J. Int..

[16] Trasatti E (2008). The 2004–2006 uplift episode at Campi Flegrei caldera (Italy): constraints from SBAS-DInSAR ENVISAT data an Bayesian source inference. Geophys. Res. Lett..

[17] Bagnardi M, Amelung F (2012). Space-geodetic evidence for multiple magma reservoirs and subvolcanic lateral intrusions at Fernandina Volcano, Galapagos Islands. J. Geophys. Res..

[18] Blakely, R. J. Potential theory in gravity and magnetic applications. Cambridge University Press (1996).

[19] Love AEH (1906). A treatise on the mathematical theory of elasticity.

[20] Sadd, M. H. Elasticity: theory, applications and numerics. Elsevier (2005).

[21] Mogi K (1958). Relation between eruptions of various volcanoes and the deformations of ground surface around them. Bull. Earthquake Res..

[22] Milano, M., Fedi, M. &Fairhead, J. D. The deep crust beneath the Trans-European Suture Zone from a multiscale magnetic model. *J. Geophys. Res. Solid Earth*, **121** (2016).

[23] Fedi M, Florio G, Quarta TAM (2009). Multiridge analysis of potential fields: geometric method and reduced Euler deconvolution. Geophysics.

[24] Fedi M (2007). DEXP: a fast method to determine the depth and the structural index of potential fields sources. Geophysics.

[25] Mann D, Freymueller J, Lu Z (2002). Deformation associated with the 1997 eruption of Okmok volcano, Alaska. J. Geophys. Res..

[26] Lu Z, Masterlark T, Dzurisin D (2005). Interferometric synthetic aperture radar study of Okmok volcano, Alaska, 1992-2003: magma supply dynamics and postemplacement lava flow deformation. J. Geophys. Res..

[27] Masterlark T, Haney M, Dickinson H, Fournier T, Searcy C (2010). Rheologic and structural controls on the deformation of Okmok volcano. J. Geophys. Res..

[28] Masterlark T (2012). Nonlinear estimation of geometric parameters in FEMs of volcano deformation: Integrating tomography models and geodetic data for Okmok volcano, Alaska. J. Geophys. Res..

[29] Tizzani P (2007). Surface deformation of Long Valley caldera and Mono Basin, California, investigated with the SBAS-InSAR approach. Rem. Sens. Env..

[30] Baranov, W. Potential Fields and their Transformations in Applied Geophysics. Geopublication Associates (1975).

[31] Ridsdill-Smith, T. A. Wavelet design of time-varying filters. Signal Process. App. (1999).

[32] Fedi M, Florio G, Paoletti V (2015). MHODE: a local-homogeneity theory for improved source-parameter estimation of potential fields. Geophys. J. Int..

[33] Thompson DT (1982). EULDPH: A new technique for making computer-assisted depth estimates from magnetic data. Geophysics.

[34] Reid AB, Allsop JM, Granser H, Millett AJ, Somerton IW (1990). Magnetic interpretation in three dimensions using Euler deconvolution. Geophysics.

[35] Finney B (2008). Magmatic Differentiation at an Island-arc Caldera: Okmok Volcano, Aleutian Islands, Alaska. Journal of Petrology.

[36] Fournelle, J.H., Marsh, B. D. & Myers, J.D. Age, character, and significance of Aleutian arc volcanism. The Geology of Alaska: Geological Society of America, 687–722.

[37] Byers, F. M. Investigations of Alaskan Volcanoes. Geol. Sur. Bull., 1028-L (1959).

[38] Miller, T. P. *et al*. Catalog of the historically active Volcanoes of Alaska (1998).

[39] Larsen J (2009). Eruption of Alaska Volcano Breaks Historic Pattern. EOS.

[40] De Luca C (2015). An On-Demand Web Tool for the Unsupervised Retrieval of Earth’s Surface Deformation from SAR Data: The P-SBAS Service within the ESA G-POD Environment. Remote Sens..

[41] De Luca C, Zinno I, Manunta M, Lanari R, Casu F (2017). Large areas surface deformation analysis through a cloud computing P-SBAS approach for massive processing of DInSAR time of DInSAR time series. Remote Sens. Environ..

