# Supplementary material for: Multiridge Method for Studying Ground-Deformation Sources: Application to Volcanic Environments
Source: Sci Rep. 2018 Sep 7;8:13420. doi: 10.1038/s41598-018-31841-4 (PMC6128938; doi:10.1038/s41598-018-31841-4)
Supplement: Supplementary file 1 — Supplementary information [file 41598_2018_31841_MOESM1_ESM.docx]

Supplementary Information for “**MULTIRIDGE METHOD FOR STUDYING GROUND-DEFORMATION SOURCES: APPLICATION TO VOLCANIC ENVIRONMENTS”**

Castaldo R.^1^, Barone A. ^2^, Fedi M.^2^, Tizzani P.^1,*^

(1) National Research Council (CNR) – Institute for the Electromagnetic Sensing of the Environment (IREA). Via Diocleziano, 328 – 80124 Napoli.

(2) Department of Earth, Environmental and Resources Science, University of Naples “Federico II”. Monte Sant’Angelo (L building), Via Cinthia 21 – 80126 Napoli.

[*tizzani.p@irea.cnr.it](mailto:*tizzani.p@irea.cnr.it) (Tizzani P.)

The Supplementary Information consists of four multi-panel figures. In particular:

**Figure S1. Mogi model vertical component: Upward continuation results.**

**Figure S2. LOS-projected Mogi model deformation field: constant reduction level procedure.**

**Figure S3. LOS-projected Mogi model: constant reduction level results.**

**Figure S4. Spherical source embedded in a heterogeneous medium: model setting.**


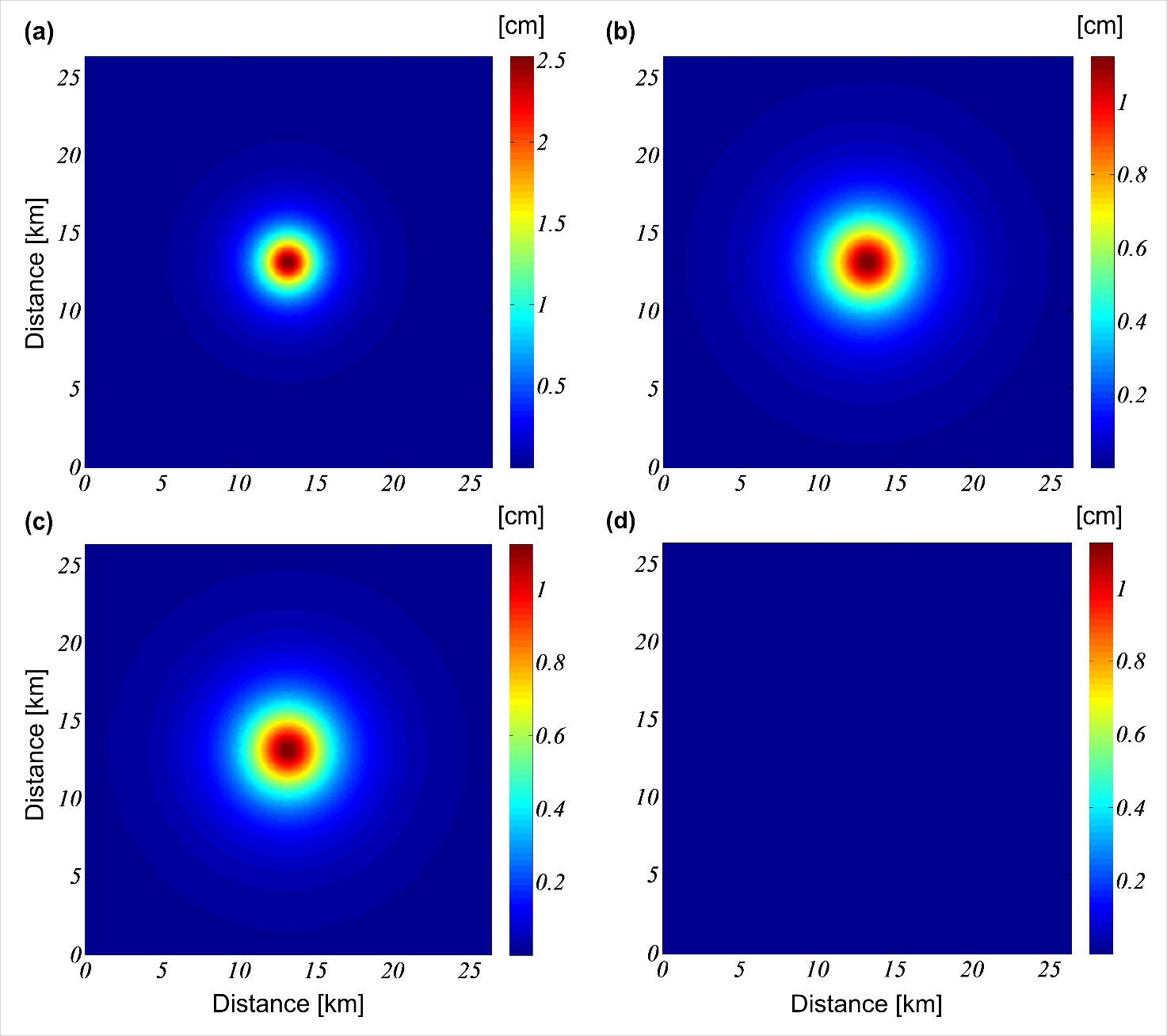
**Figure S1. Mogi model vertical component: Upward continuation results.** (a) Map of the modeled vertical component of deformation field generated by a Mogi source located at 2 km of depth and calculated on a flat surface level of $z=0$; (b) Upward-continued field at 1 km. (c) Map of the modeled vertical component of deformation field generated by a Mogi source, located at 3 km of depth and calculated on a flat surface level of $z=0$. (d) Differences between (b) and (c) maps. The model parameters are: radius = 0.3 [km], ΔP = 5 [MPa], G = 1 [GPa], ν = 0.25 [-].


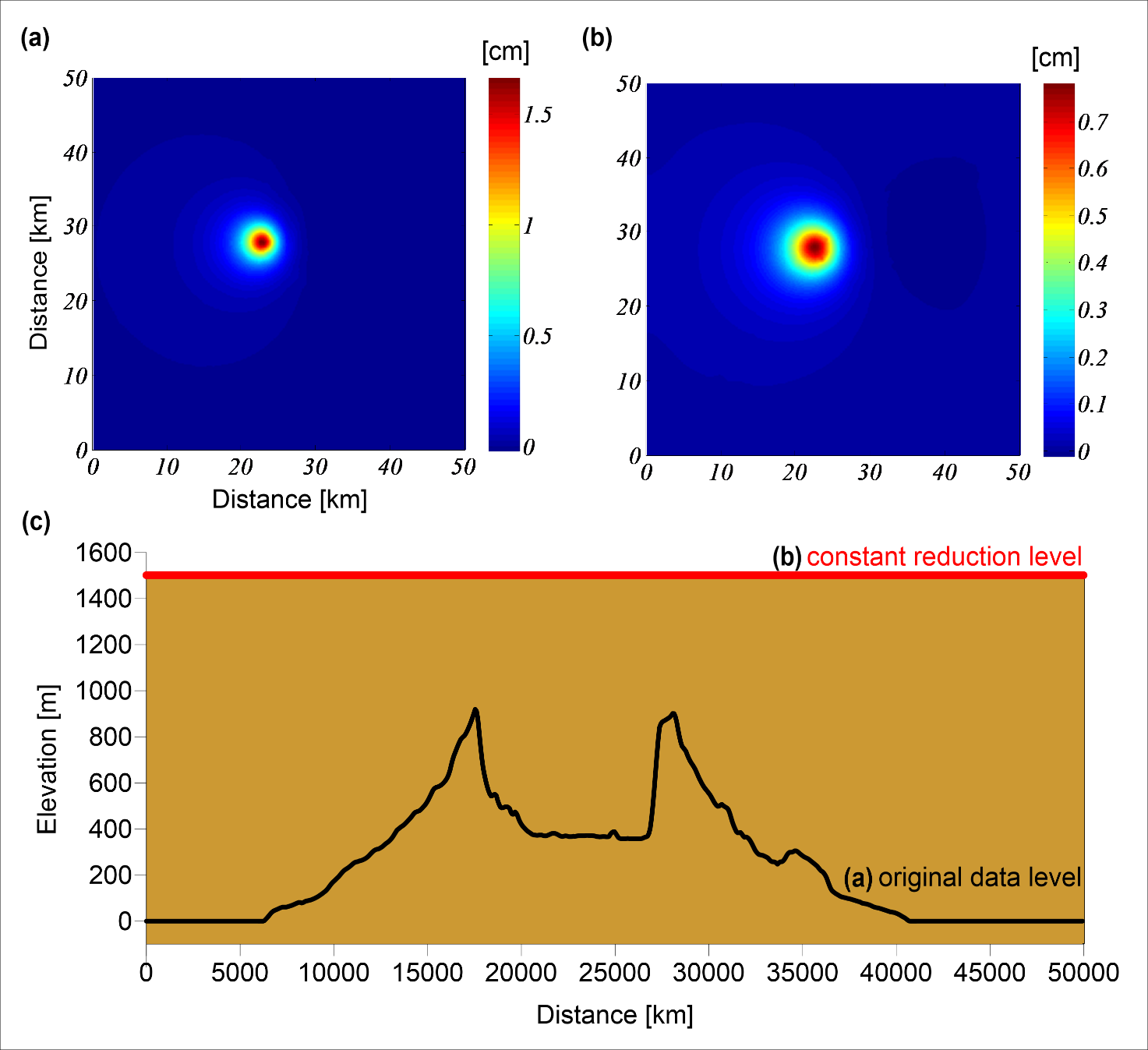


**Figure S2. LOS-projected Mogi model deformation field: constant reduction level procedure.** (a) Map of the modeled ascending LOS calculated on the Okmok volcano topographic surface. (b) Ascending LOS deformation map reduced to the flat surface: 1.5 km a.s.l. (c) Sketch showing the different data level between the case (a) and (b). The model parameters are: radius = 0.3 [km], ΔP = 5 [MPa], G = 1 [GPa], ν = 0.25 [-]. The used mean LOS vector ([0.346, -0.081, 0.935]) is related to a 23° satellite look angle.

**
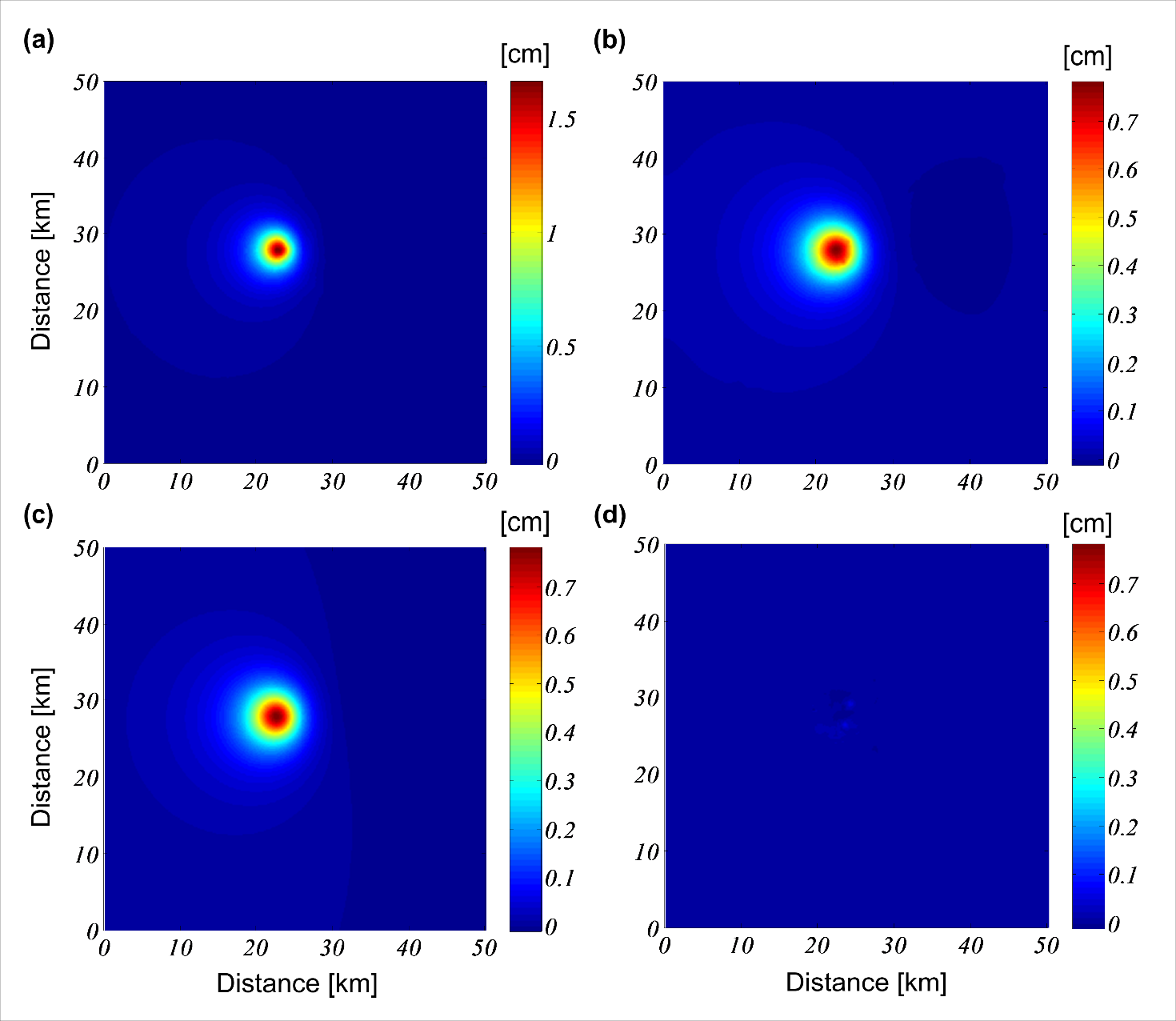
**

**Figure S3. LOS-projected Mogi model: constant reduction level results.** (a) Map of the modelled Ascending LOS deformation calculated on Okmok volcano topography. The Mogi source is located at 2 km depth. (b) Ascending LOS deformation map reduced to the constant level; the flat surface is at 1.5 km a.s.l. (see Figure S3). (c) Ascending LOS modelled deformation field generated by a Mogi source located at 3.5 km depth and measured on a flat surface. (d) Differences between (b) and (c). The model parameters are: radius = 0.3 [km], ΔP = 5 [MPa], G = 1 [GPa], ν = 0.25 [-]. The used mean LOS vector ([0.346, -0.081, 0.935]) is related to a 23° satellite look angle.


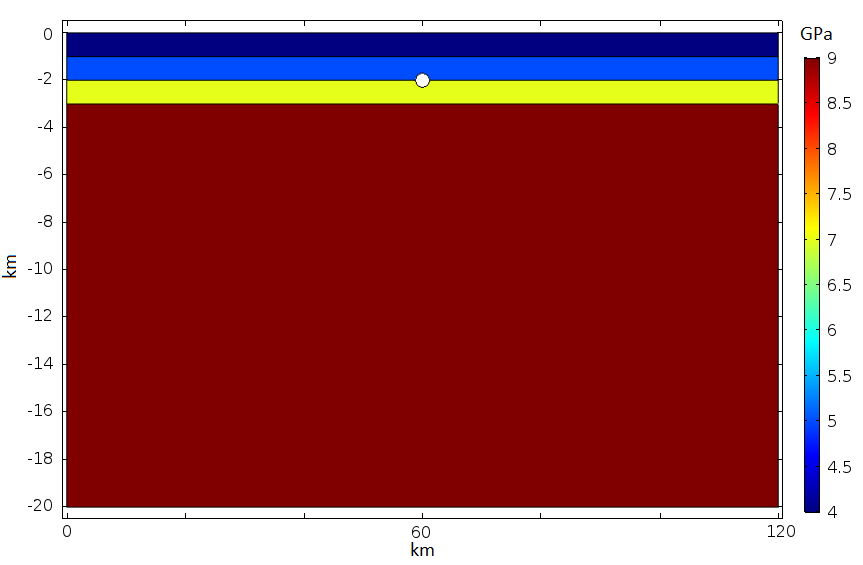


**Figure S4. Spherical source embedded in a heterogeneous medium: model setting.** 2D section of the heterogeneous 3D FE (finite element) model used for the performed test, shown in Figure 3. The spherical source is embedded in a multilayer space characterized by increasing of the shear modulus with the depth. In particular, the model parameters are: depth of the source center ($z_{0}$) $=2$ [km], E-W position of the source ($x_{0}$) $=60$ [km], N-S position of the source ($y_{0}$) $=60$ [km], radius of the source ($A$) $=0.3$ [km], overpressure of the source ($\Delta P$) $=10$ [Mpa], Poisson’s coefficient of the medium ($\nu$) $=0.25$ [-]. The medium is characterized by the shear modulus ($G$) varying with depth as follow: from 0 to 1 km $G_{1}=4$ [GPa]; from 1 to 2 km $G_{2}=5$ [GPa]; from 2 to 3 km $G_{3}=7$ [GPa] and from 3 km to 20 km $G_{4}=9$ [GPa].
